# Factors Associated With Prolonged Emergency Department (ED) Length of Stay (LOS) at a Tertiary Care Hospital in Nepal: A Cross‐Sectional Study

**DOI:** 10.1155/emmi/8919991

**Published:** 2026-10-06

**Authors:** Pratisha Pradhan, Kabita Hada Batajoo, Alok Kumar, K. C. Bhushan Bikram, Sneha Pradhananga, Trishna Shrestha

**Affiliations:** ^1^ Department of General Practice and Emergency Medicine KIST Medical College Teaching Hospital Imadol Lalitpur Bagmati Province Nepal

**Keywords:** emergency, length of stay, overcrowding, prolonged length of stay

## Abstract

**Background:**

Emergency department (ED) length of stay (LOS) is an important indicator of healthcare efficiency and patient flow. The duration of ED stay is influenced by a complex interaction of patient‐related, clinical and system‐level factors. Prolonged LOS (> 4 h) contributes to ED overcrowding and adverse patient outcomes. However, limited data exist regarding factors influencing ED LOS in Nepal.

**Method:**

A descriptive cross‐sectional study was conducted in the ED of KIST Medical College and Teaching Hospital (MCTH), a tertiary care hospital in Lalitpur, Nepal, from May 15, 2025, to May 30, 2025. All patients presenting to the ED during the study period were eligible for inclusion. Consecutive patient records were reviewed. ED LOS was defined as the time from patient arrival to ED exit. LOS was summarized using median and interquartile range (IQR). Group differences were assessed using the Mann–Whitney U and Kruskal–Wallis tests. Variables with *p* < 0.20 in univariate analysis were entered into a multivariable logistic regression model to identify factors independently associated with prolonged ED LOS (> 4 h). Adjusted odds ratios (aORs) with 95% confidence intervals (CIs) were reported.

**Results:**

A total of 395 patients were included. The median ED LOS was 3.58 h (IQR 2.42–5.00). A total of 161 (40.8%) experienced prolonged ED LOS, while 234 (59.2%) had a LOS of ≤ 4 h. In univariate analysis, age, triage category, number of specialty consultations, payment method and requirement for advanced investigations were significantly associated with prolonged ED LOS (*p* < 0.05). On multivariable logistic regression analysis, increasing age, time of presentation and number of specialty consultations were independent predictors of prolonged ED LOS. Patients requiring single specialty consultation had higher odds of prolonged LOS (aOR 3.12; 95% CI 1.34–7.28), while those requiring multiple consultations had markedly higher odds (aOR 13.41; 95% CI 4.47–40.20). Presentation during night shift was associated with lower odds of prolonged LOS compared with morning shift (aOR 0.29; 95% CI 0.16–0.52).

**Conclusions:**

ED LOS in this tertiary care hospital was primarily influenced by patient age, time of presentation, consultation processes and administrative factors. Strengthening interdepartmental consultation processes and improving ED workflow efficiency may help reduce prolonged ED stay and overcrowding.

## 1. Introduction

Emergency department (ED) length of stay (LOS) is defined as the time interval between a patient’s arrival to the ED (“time‐in”) and the moment the patient physically leaves the ED (“time‐out”) [1]. The duration of ED stay varies widely across healthcare institutions due to differences in infrastructure, staffing patterns, patient volume and operational workflows [2]. Timeliness of care is widely regarded as a key indicator of ED performance and quality of care [3, 4]. Prolonged LOS is defined as an ED stay of more than 4 h as per the National Health Service (NHS) [5]. It is frequently associated with ED overcrowding, a situation in which the demand for emergency services exceeds the available capacity of the department to provide timely care [6].

The duration of ED stay is influenced by a complex interaction of patient‐related, clinical and system‐level factors. Patient characteristics, such as advanced age and comorbidities, may increase the complexity of clinical management and prolong evaluation. In addition, high‐acuity presentations, the need for extensive diagnostic investigations and delays in specialty consultations can contribute to longer ED stays. System‐level factors, including administrative processes, insurance verification, limited availability of diagnostic services during off‐hours and shortages of inpatient beds, also play an important role in prolonging LOS [6–8]. Prolonged ED LOS has been associated with several adverse consequences, including delays in definitive treatment, increased morbidity and mortality, reduced patient satisfaction and decreased efficiency of healthcare providers [9, 10].

In response to the challenges posed by ED overcrowding, several healthcare systems have implemented time‐based performance targets aimed at improving patient flow. One notable example is the “4‐h rule” introduced in the United Kingdom in 2004, which required that the majority of ED patients be assessed, treated and either discharged or admitted within four hours of arrival [5, 11]. Evidence from multiple studies suggests that prolonged ED LOS is associated with poorer clinical outcomes, including higher mortality rates, longer inpatient hospital stays and reduced overall healthcare productivity [11–14].

In Nepal, only a limited number of studies have examined prolonged ED LOS [1, 3, 7, 15, 16]. Existing research has largely focused on describing ED LOS, patient flow or disposition patterns, with limited evaluation of factors independently associated with prolonged ED LOS. Therefore, this study aimed to determine the median ED LOS, compare the characteristics of patients with prolonged and nonprolonged ED LOS using a predefined 4‐h cutoff and identify independent patient‐ and system‐related predictors of prolonged ED LOS in a tertiary care hospital.

## 2. Methods

### 2.1. Study Design and Setting

A descriptive cross‐sectional study was conducted in the ED of KIST Medical College and Teaching Hospital (KIST MCTH), Imadol, Lalitpur, Nepal. The study included all patients presenting to the ED during the study period, May 15–May 30, 2025. Ethical approval for the study was obtained from the Institutional Review Committee (IRC) of KIST MCTH (Ref: 8182‐3008024‐12).

Normally, patients presenting to the ED are initially received at the triage area, where trained intern or nursing staff perform rapid assessment, including documentation of chief complaints, duration of symptoms and vital parameters (pulse rate, blood pressure, respiratory rate, oxygen saturation, temperature and mental status). Based on clinical urgency, patients are categorized according to a standardized triage system, the Australasian Triage Scale (ATS) [17], and directed to appropriate care areas. According to this scale, the recommended maximum waiting times for medical assessment and initiation of treatment are as follows: immediate for ATS Category 1, within 10 min for Category 2, within 30 min for Category 3, within 60 min for Category 4 and within 120 min for Category 5.

Following triage, patients undergo primary evaluation by the on‐duty consultant, medical officer (MO) or ED resident. Initial stabilization and resuscitation are performed concurrently where indicated. Diagnostic investigations, including laboratory tests and imaging, are ordered based on clinical assessment.

Patients are then managed in the ED with ongoing monitoring, reassessment and specialist consultations when necessary. Based on clinical condition and response to treatment, a disposition decision is made, including discharge, admission, referral or discharge on request (DOR). The total ED LOS is defined as the time interval between a patient’s arrival to the ED (“time‐in”) and patient exit from the ED (“time‐out”).

### 2.2. Study Population and Sampling

All patients presenting to the ED during the study period were eligible for inclusion. Data were collected prospectively from paper‐based ED records, without direct patient interaction; therefore, individual consent was not required. This approach was reviewed and approved by the IRC. Records with incomplete or missing key variables required for calculating ED LOS were excluded from the analysis.

### 2.3. Sample Size Calculation

All eligible patients presenting to the ED during the study period were included using consecutive sampling. A total of 427 records were collected but 32 were excluded due to missing variables.

### 2.4. Study Variables and Definitions

ED LOS was defined as the time interval between patient arrival to the ED (“time‐in”) and the time of departure from the ED (“time‐out”).

Consultation time was defined as the time difference between the consultation request made by the emergency physician and the time the consultant reviewed the patient, as recorded in the ED documentation.

Additional process intervals were also recorded, including

• Arrival to triage
• Triage to first clinical assessment
• Arrival to consultation request
• Consultation request to consultation
• Consultation to disposition decision
• Disposition decision to ED exit

### 2.5. Data Collection

Data were collected prospectively from the ED records as patients arrived, without direct patient interaction; therefore, individual consent was not required. This approach was reviewed and approved by the IRC. Data were then entered into a predesigned Microsoft Excel data collection sheet. Collected variables included patient demographic characteristics, triage category, referral status, investigations performed, consultations requested, payment method and final patient disposition.

### 2.6. Statistical Analysis

Data were analysed using the Statistical Package for Social Sciences (SPSS) Version 18. Continuous variables were assessed for normality using the Shapiro–Wilk test. Since ED LOS was not normally distributed, data were summarized as median (IQR). The Mann–Whitney *U* and Kruskal–Wallis tests were used for univariate comparisons. Variables with *p* < 0.20 in univariate analysis were entered into a multivariable logistic regression model to identify independent factors associated with prolonged ED LOS (> 4 h). Adjusted odds ratios with 95% confidence intervals were reported. Statistical significance was defined as *p* < 0.05.

## 3. Results

A total of 395 patient records were included in the analysis. The overall median ED LOS was 3.58 h (IQR: 2.42–5.00).

### 3.1. Patient Characteristics and ED LOS

The distribution of ED LOS according to patient characteristics is shown in Table 1.

**TABLE 1:** Baseline characteristics and median ED LOS (in hours).

Characteristics	*n* (%)	Median LOS (in hrs)	IQR	*p* value
Age				
< 1 year	5 (1.3)	3.33	2.42–3.83	< 0.001^∗^
1–15 years	67 (17.0)	2.83	1.77–3.54	
15–65 years	253 (64.0)	3.58	2.42–4.83	
≥ 65 years	70 (17.7)	5.21	3.58–6.50	
Sex				
Male	197 (49.9)	3.50	2.33–4.83	0.29^∗∗^
Female	198 (50.1)	3.67	2.58–5.08	
Prehospital treatment				
Yes	10 (2.5)	4.17	2.87–5.17	0.43^∗∗^
No	385 (97.5)	3.58	2.42–5.00	
Referred in				
Yes	22 (5.6)	4.83	2.75–6.25	0.08^∗∗^
No	373 (94.4)	3.53	2.42–4.83	
Revisit				
Yes	1 (0.3)	—	—	—
No	394 (99.7)	3.58	2.42–5.00	
Shift of the day				
Morning	119 (30.1)	4.25	3.33–6.35	< 0.001^∗^
Evening	118 (29.9)	3.75	2.17–5.60	
Night	158 (40.0)	3.07	2.08–4.00	
Triage category				
I	18 (4.6)	3.88	2.42–4.58	< 0.001^∗^
II	70 (17.7)	4.83	3.33–6.50	
III	305 (77.2)	3.33	2.20–4.67	
IV	2 (0.5)	1.36	1.13–1.58	
V	0 (0)	—	—	
Payment method				
Self‐payment	300 (75.9)	3.42	2.25–4.75	< 0.001^∗∗^
Insurance	95 (24.1)	4.23	3.17–5.79	
Investigations sent				
First setting from ED	351 (88.8)	3.58	2.50–5.00	< 0.005^∗^
Added after departmental consultation	9 (2.3)	4.00	2.25–5.17	
Advanced imaging (CT/MRI)	24 (6.1)	3.83	2.39–5.71	
Not needed	3 (0.8)	1.00	0.92–1.87	
Refused	8 (2.0)	1.62	1.23–2.85	
Consultations				
Emergency department	55 (13.9)	2.67	2.00–3.54	< 0.001^∗^
Single department	290 (73.4)	3.52	2.42–4.83	
Multiple departments	50 (12.7)	5.13	4.08–7.33	
Outcome				
Discharge	215 (54.5)	3.25	2.08–4.46	< 0.001^∗^
DOPR	81 (20.5)	3.58	2.58–4.75	
Ward admission	70 (17.7)	5.21	3.25–7.50	
ICU	13 (3.3)	4.58	3.75–5.83	
HDU	6 (1.5)	4.19	3.33–4.42	
PICU	4 (1.0)	3.33	2.29–3.58	
Semicabin	2 (0.5)	6.25	5.67–6.83	
Cabin	2 (0.5)	2.83	2.50–3.17	
Shift to operation theatre	0 (0)	—	—	
Shift to Cath lab	2 (0.5)	0.93	0.67–1.20	

^∗^Kruskal–Wallis.

^∗∗^Mann–Whitney *U*.

ED LOS differed significantly across age categories (*p* < 0.001). Older patients experienced progressively longer ED LOS, with those aged ≥ 65 years demonstrating the longest median stay. No significant difference in ED LOS was observed between male and female patients (*p* 0.29).

Patients who received prehospital treatment or were referred from other healthcare facilities had slightly longer median ED LOS; however, these differences were not statistically significant (*p* = 0.43 and *p* = 0.08, respectively).

### 3.2. Operational Factors Associated With ED LOS

ED LOS varied significantly according to the time of presentation (*p* < 0.001). Patients presenting during the morning shift experienced the longest median LOS, followed by those presenting during the evening shift, while the night shift had the shortest LOS.

A significant difference in LOS was also observed across triage categories (*p* < 0.001), with higher‐acuity patients demonstrating longer ED stays.

Patients utilizing insurance‐based payment methods had significantly longer ED LOS compared with those paying out‐of‐pocket (*p* < 0.001).

Similarly, the type and timing of investigations influenced LOS. Patients requiring advanced imaging, such as CT or MRI or those undergoing additional investigations following specialty consultation, had significantly longer LOS compared with patients managed with initial investigations from the ED (*p* < 0.005).

### 3.3. Consultations and Patient Disposition

ED LOS increased with the number of specialty consultations requested (*p* < 0.001). Patients managed exclusively by the ED had the shortest median LOS, whereas patients requiring multiple specialty consultations had the longest stays.

Patient disposition was also significantly associated with ED LOS (*p* < 0.001). Patients admitted to inpatient wards or critical care units had longer ED stays compared with those discharged from the ED.

### 3.4. Univariate Analysis

Univariate analysis of factors associated with prolonged ED LOS (> 4 h) is presented in Table 2. Of the 395 patients included in the study, 161 (40.8%) experienced prolonged ED LOS, while 234 (59.2%) had a LOS of ≤ 4 h. Age, referral status, shift of presentation, payment method, number of consultations, triage category, primary consulting department and patient disposition were significantly associated with prolonged ED LOS (all *p* < 0.05). In contrast, sex, prehospital treatment, revisit status and investigations performed were not significantly associated with prolonged ED LOS (*p* > 0.05).

**TABLE 2:** Univariate analysis of factors associated with prolonged ED LOS.

Characteristics	*n* (%)	Not prolonged LOS *n* (%)	Prolonged LOS *n* (%)	*p* value^∗^
		234 (59.2)	161 (40.8)	
Age group				
• < 1 year	5 (1.3)	5 (100.0)	0 (0.0)	< 0.001
• 1–15 years	67 (17.0)	56 (83.6)	11 (16.4)	
• 15–65 years	253 (64.0)	152 (60.1)	101 (39.9)	
• ≥ 65 years	70 (17.7)	21 (30.0)	49 (70.0)	
Sex				
• Male	197 (49.9)	118 (59.9)	79 (40.1)	0.791
• Female	198 (50.1)	116 (58.6)	82 (41.4)	
Prehospital treatment				
• Yes	10 (2.5)	5 (50.0)	5 (50.0)	0.547
• No	385 (97.5)	229 (59.5)	156 (40.5)	
Referred in				
• Yes	22 (5.6)	8 (36.4)	14 (63.6)	0.025
• No	373 (94.4)	226 (60.6)	147 (39.4)	
Revisit				
• Yes	1 (0.3)	1 (100.0)	0 (0.0)	0.406
• No	394 (99.7)	233 (59.1)	161 (40.9)	
Shift of the day				
• Morning	119 (30.1)	49 (41.2)	70 (58.8)	< 0.001
• Evening	118 (29.9)	66 (55.9)	52 (44.1)	
• Night	158 (40.0)	119 (75.3)	39 (24.7)	
Payment method				
• Self‐payment	300 (75.9)	190 (63.3)	110 (36.7)	0.003
• Insurance	95 (24.1)	44 (46.3)	51 (53.7)	
Investigations sent				
• First setting from ED	351 (88.8)	202 (57.5)	149 (42.5)	0.081
• Added after departmental consultation	9 (2.3)	6 (66.7)	3 (33.3)	
• Advanced imaging (CT/MRI)	24 (6.1)	15 (62.5)	9 (37.5)	
• Not needed	3 (0.8)	3 (100.0)	0 (0.0)	
• Refused	8 (2.0)	8 (100.0)	0 (0.0)	
Consultations				
• Emergency department	55 (13.9)	47 (85.5)	8 (14.5)	< 0.001
• Single department	290 (73.4)	175 (60.3)	115 (39.7)	
• Multiple departments	50 (12.7)	12 (24.0)	38 (76.0)	
Triage category				
• I	18 (4.6)	10 (55.6)	8 (44.4)	< 0.001
• II	70 (17.7)	26 (37.1)	44 (62.9)	
• III	305 (77.2)	196 (64.3)	109 (35.7)	
• IV	2 (0.5)	2 (100.0)	0 (0.0)	
• V	0 (0)	0 (0.0)	0 (0.0)	
Primary department consultations				
• ED	62 (15.7)	52 (83.9)	10 (16.1)	< 0.001
• Medicine	132 (33.4)	39 (29.5)	93 (70.5)	
• Surgery	49 (12.4)	27 (55.1)	22 (44.9)	
• Orthopaedics	57 (14.4)	50 (87.7)	7 (12.3)	
• Paediatrics	39 (9.9)	29 (74.4)	10 (25.6)	
• Obstetrics and gynaecology	14 (3.5)	13 (92.9)	1 (7.1)	
• Dermatology	3 (0.8)	2 (66.7)	1 (33.3)	
• ENT	11 (2.8)	5 (45.5)	6 (54.5)	
• Ophthalmology	1 (0.3)	1 (100.0)	0 (0.0)	
• Psychiatry	24 (6.1)	13 (54.2)	11 (45.8)	
• OMFS	3 (0.8)	3 (100.0)	0 (0.0)	
Outcome				
• Discharge	215 (54.5)	144 (67.0)	71 (33.0)	< 0.001
• DOPR	81 (20.5)	51 (63.0)	30 (37.0)	
• Ward admission	70 (17.7)	24 (34.3)	46 (65.7)	
• ICU	13 (3.3)	5 (38.5)	8 (61.5)	
• HDU	6 (1.5)	2 (33.3)	4 (66.7)	
• PICU	4 (1.0)	4 (100.0)	0 (0.0)	
• Semicabin	2 (0.5)	0 (0.0)	2 (100.0)	
• Cabin	2 (0.5)	2 (100.0)	0 (0.0)	
• Shift to Cath lab	2 (0.5)	2 (100.0)	0 (0.0)	

^∗^Chi‐square test.

### 3.5. Multivariable Logistic Regression

Variables meeting the inclusion criterion in univariate analysis were entered into the multivariable logistic regression model. In multivariable logistic regression analysis, several factors were independently associated with prolonged ED LOS (Table 3). Age was a significant predictor, with patients aged ≤ 15 years showing significantly lower odds of prolonged ED LOS compared with the reference group (15–65 years) (aOR 0.29; 95% CI 0.14–0.61; *p* = 0.001), while those aged ≥ 65 years had higher odds (aOR 1.99; 95% CI 1.04–3.81; *p* = 0.038).

**TABLE 3:** Multivariable analysis of factors associated with prolonged ED LOS.

Variable categories	cOR (95% CI)	*p* value	aOR (95% CI)	*p* value
Age				< 0.001
• < 15 years	0.27 (0.14–0.54)	< 0.001	0.29 (0.14–0.61)	0.001
• 15–65 years	1.00 (Reference)	—	1.00 (Reference)	—
• ≥ 65 years	3.51 (1.99–6.21)	< 0.001	1.99 (1.04–3.81)	0.038
Sex				
• Male	1.00 (Reference)	—	1.00 (Reference)	—
• Female	1.06 (0.71–1.58)	0.791	1.07 (0.67–1.72)	0.783
Referred in				
• Yes	2.69 (1.10–6.57)	0.03	1.81 (0.64–5.14)	0.267
• No	1.00 (Reference)	—	1.00 (Reference)	—
Shift of the day				< 0.001
• Morning	1.00 (Reference)	—	1.00 (Reference)	—
• Evening	0.55 (0.33–0.92)	0.024	0.58 (0.32–1.05)	0.074
• Night	0.23 (0.14–0.38)	< 0.001	0.29 (0.16–0.52)	< 0.001
Payment method				
• Self‐payment	1.00 (Reference)	—	1.00 (Reference)	—
• Insurance	2.00 (1.26–3.19)	0.004	1.33 (0.75–2.35)	0.328
Diagnostic investigations				
• Routine or no investigations	1.00 (Reference)	—	1.00 (Reference)	—
• Additional/advanced imaging	0.82 (0.39–1.71)	0.592	0.62 (0.25–1.56)	0.310
Consultations				< 0.001
• ED only	1.00 (Reference)	—	1.00 (Reference)	—
• Single department	3.86 (1.76–8.47)	0.001	3.12 (1.34–7.28)	0.008
• Multiple departments	18.60 (6.90–50.15)	< 0.001	13.41 (4.47–40.20)	< 0.001
Triage category				0.324
• I	1.45 (0.56–3.79)	0.445	1.08 (0.34–3.44)	0.892
• II	3.07 (1.79–5.27)	< 0.001	1.66 (0.85–3.23)	0.135
• III + IV	1.00 (Reference)	—	1.00 (Reference)	—
Outcome				0.153
• Discharged	1.00 (Reference)	—	1.00 (Reference)	—
• Ward and other admissions	3.31 (1.96–5.59)	< 0.001	1.82 (0.99–3.33)	0.054
• Critical care admission	2.11 (0.90–4.94)	0.087	1.08 (0.36–3.23)	0.891

*Note:* cOR, crude odds ratio.

Abbreviations: aOR, adjusted odds ratio; CI, confidence interval.

The number of consultations demonstrated the strongest association with prolonged ED LOS. Patients requiring consultation from a single specialty had approximately threefold higher odds of prolonged LOS compared with those managed solely in the ED (aOR 3.12; 95% CI 1.34–7.28; *p* = 0.008), while those requiring multiple specialty consultations had markedly increased odds (aOR 13.41; 95% CI 4.47–40.20; *p* < 0.001). Presentation during night shifts was associated with significantly lower odds of prolonged ED LOS compared with morning shifts (aOR 0.29; 95% CI 0.16–0.52; *p* < 0.001). Other variables, including sex, referral status, payment method, investigations performed, triage category and disposition, were not independently associated with prolonged ED LOS after adjustment.

### 3.6. ED Process Intervals

Analysis of ED process intervals is presented in Table 4. The arrival‐to‐triage and triage‐to‐first clinical assessment intervals demonstrated minimal delays, with median times of 0 min and 1 min, respectively.

**TABLE 4:** ED process interval timings at ED (*N* = 395).

ED process interval	Median time (in minutes)	IQR
Arrival to triage	0.00	0.00–0.00
Triage to first clinical assessment	1.00	1.00–2.00
Arrival to payment	19.00	14.00–27.90
Arrival to consultation request^∗^	95.00	45.00–150.00
Consultation request to consultation^∗^	20.00	14.00–40.00
Consultation to disposition decision^∗^	75.00	45.00–140.00
Disposition decision to ED exit	5.00	0.00–5.00
Arrival to exit (total ED LOS)	215.00	145.00–300.00

^∗^ *N* = 333 (excluding ED dispatch).

Longer delays were observed in consultation‐related intervals. The median time from arrival to consultation request was 95 min (IQR 45–150), while the interval between consultation and disposition decision was 75 min (IQR 45–140). The median total ED LOS was 215 min (IQR 145–300).

### 3.7. Primary Consultation Department and ED LOS

ED LOS varied significantly according to the primary consulting department (*p* < 0.001), as shown in Table 5. Patients referred to the Medicine and ENT departments had the longest median LOS. In contrast, patients referred to Orthopaedics and Obstetrics and Gynaecology had relatively shorter stays in the ED.

**TABLE 5:** LOS at ED according to primary consultation departments and the process timings.

Primary consultation department			Consultation call time to consultation time (in minutes)		*p* value^∗^	Consultation time to dispatch time (in minutes)		*p* value^∗^	LOS (in hours)		*p* value^∗^
*N* = 395	*n*	%	Median	IQR		Median	IQR		Median	IQR	
ED	62	15.7	N/A		< 0.001	N/A		< 0.001	2.71	2.00–3.58	< 0.001
Medicine	132	33.4	30.00	17.50–70.00		115.00	62.50–170.00		5.00	3.90–6.46	
Surgery	49	12.4	30.00	15.00–30.00		75.00	40.00–110.00		3.83	3.08–5.25	
Orthopaedics	57	14.4	15.00	10.00–20.00		55.00	40.00–90.00		2.03	1.58–2.92	
Paediatrics	39	9.9	15.00	7.50–22.50		60.00	35.00–95.00		3.08	2.25–4.04	
OBG	14	3.5	12.50	10.00–20.00		66.50	50.00–75.00		2.62	2.08–3.50	
Dermatology	3	0.8	30.00	30.00–32.50		30.00	25.00–60.00		3.17	3.04–3.62	
ENT	11	2.8	25.00	12.50–30.00		130.00	75.00–190.00		4.08	2.17–5.08	
Ophthalmology	1	0.3	—	—		—	—		—	—	
Psychiatry	24	6.1	20.00	15.00–30.00		62.50	37.50–75.00		3.88	3.07–4.39	
OMFS	3	0.8	40.00	27.50–42.50		120.00	82.50–130.00		3.08	2.58–3.18	

^∗^Kruskal–Wallis test.

## 4. Discussion

This study evaluated ED LOS and factors associated with prolonged ED stay in a tertiary care hospital in Nepal. The median ED LOS was 3.58 h (IQR 2.42–5.00), with approximately two‐fifths of patients (41%) experiencing prolonged ED LOS (> 4 h). Multivariable logistic regression identified increasing age, time of presentation and the number of specialty consultations as independent predictors of prolonged ED LOS. In contrast, although triage category, payment method, investigations performed and patient disposition were associated with prolonged ED LOS in univariate analyses, these associations were no longer significant after adjustment for potential confounders. Process interval analysis further demonstrated that the greatest delays occurred during consultation‐related phases of patient management, highlighting downstream clinical workflow as the principal contributor to prolonged ED stay.

The median ED LOS in the present study is comparable to findings reported from other tertiary care hospitals in Nepal. A study conducted at Kathmandu Medical College (KMC) reported a mean ED LOS of 3.18 h, reflecting similar patient flow dynamics in tertiary EDs in the country [1]. Although direct comparisons should be interpreted cautiously because of differences in study design and summary measures, the overall duration of ED stay appears consistent within the Nepalese context. On the contrary, studies from healthcare systems experiencing severe access block and inpatient bed shortages have reported substantially longer ED stays, reflecting differences in hospital capacity and patient flow dynamics [18].

Increasing age was an independent determinant of prolonged ED LOS in this study. Patients aged ≥ 65 years had significantly higher odds of prolonged ED stay, whereas patients younger than 15 years were less likely to remain in the ED for more than four hours. Older adults frequently present with multiple comorbidities, atypical clinical presentations and greater diagnostic complexity, often requiring more extensive evaluation, additional investigations and involvement of multiple specialties before disposition decisions can be made. Similar associations between increasing age and prolonged ED LOS have been reported in previous studies, emphasizing the growing impact of ageing populations on emergency care systems [4, 8, 19].

The time of patient presentation was another factor significantly influencing ED LOS. Patients presenting during the night shift had lower odds of prolonged ED stay compared with those presenting in the morning shift. The longer LOS observed during daytime hours may reflect increased patient volumes, greater demand for diagnostic services and competing hospital activities, such as outpatient clinics, elective procedures and administrative processes. These factors may contribute to delays in investigations, consultations and disposition despite the availability of a larger workforce. Similar temporal variations in ED throughput have been described in previous studies evaluating operational determinants of ED crowding [8, 19, 20].

The requirement of specialty consultations emerged as the strongest independent predictor of prolonged ED LOS. Patients requiring consultation from a single specialty had approximately threefold higher odds of prolonged ED stay, whereas those requiring consultations from multiple specialties had more than 13‐fold higher odds compared with patients managed exclusively by the ED. Each additional consultation introduces further assessment, communication, investigation review and multidisciplinary decision‐making before definitive patient disposition, thereby increasing the overall duration of ED stay. Similar findings have been reported by Hosseininejad et al. [4], who identified delayed consultant response as a major contributor to prolonged ED LOS, and by Akram et al. [21], who also reported specialty consultation as an important determinant of delayed patient disposition.

The process interval analysis further supports these findings. Initial ED processes were highly efficient, with minimal delays between patient arrival, triage and first clinical assessment. In contrast, substantially longer intervals were observed between consultation request and specialist review, as well as between consultation and disposition decision. The concordance between the multivariable regression findings and the process interval analysis suggests that consultation‐related workflow, rather than delays in initial emergency assessment, is a major contributor to prolonged ED LOS in our setting. These findings indicate that interventions aimed at improving consultation response times, strengthening communication between emergency physicians and specialty teams and streamlining interdepartmental decision‐making may have a greater impact on reducing ED LOS than interventions directed solely at front‐end ED processes.

Several variables, including triage category, payment method, investigations performed and patient disposition, were significantly associated with prolonged ED LOS in univariate analyses but were not retained as independent predictors in the multivariable model. Higher‐acuity patients, those requiring insurance‐based payment, advanced diagnostic investigations or hospital admission tended to remain longer in the ED; however, these factors may exert their influence indirectly through increased clinical complexity and the greater likelihood of requiring specialty consultation. Their loss of statistical significance after adjustment suggests that consultation burden, patient age and operational factors explain much of the observed variation in ED LOS. This distinction highlights the importance of multivariable analysis in identifying independent determinants while accounting for confounding between related clinical and system‐level factors.

Prolonged ED LOS has important implications for patient outcomes and healthcare system efficiency. Several studies have demonstrated associations between prolonged ED stay and increased morbidity, mortality and inpatient LOS [9, 12]. More recent research also suggests that longer ED stays may be associated with prolonged hospitalization and increased healthcare resource utilization [22]. Although patient outcomes were not evaluated in the present study, identifying modifiable operational factors provides an opportunity to improve patient flow and reduce ED overcrowding. Given that consultation‐related processes represented the most prominent bottleneck in this study, targeted interventions aimed at improving consultation response times, facilitating earlier decision‐making and streamlining interdepartmental coordination may substantially improve ED efficiency in similar resource‐limited settings.

This study has several limitations. First, it was conducted in a single tertiary care hospital, which may limit the generalizability of the findings to other healthcare settings. Second, the cross‐sectional design allows identification of associations but does not establish causal relationships. Third, several potentially important determinants of ED LOS, including inpatient bed availability, ED crowding status, staffing levels, consultant workload and disease severity, were not evaluated. Nevertheless, the study has important strengths. It prospectively evaluated consecutive ED presentations, incorporated both patient‐ and system‐related variables and used multivariable logistic regression to identify independent predictors of prolonged ED LOS. In addition, the inclusion of process interval analysis allowed identification of specific stages within the ED workflow where delays occurred, providing practical targets for quality improvement initiatives.

Overall, the findings indicate that prolonged ED LOS in this tertiary care hospital is influenced predominantly by operational and workflow factors rather than patient characteristics alone. Improving consultation efficiency and strengthening coordination between emergency and specialty services may therefore represent the most effective strategies for reducing ED LOS and improving patient flow in similar emergency care settings.

## 5. Conclusion

This study demonstrates that 161 (40.8%) experienced prolonged ED LOS, while 234 (59.2%) had a LOS of ≤ 4 h. ED LOS in this tertiary care hospital in Nepal was primarily influenced by system‐level workflow and coordination factors rather than patient demographics alone. Age, time of presentation and the number of specialty consultations were identified as independent predictors of prolonged ED LOS. In particular, the need for multiple specialty consultations emerged as the strongest determinant of prolonged stay, highlighting the critical role of interdepartmental communication and consultation efficiency in ED throughput.

Targeted strategies aimed at improving consultation response times, strengthening coordination between emergency and specialty services and optimizing patient flow processes may help reduce ED congestion and improve efficiency. Addressing these operational bottlenecks is essential to enhancing emergency care delivery and reducing prolonged ED stay in similar resource‐limited settings.

## Funding

The authors have nothing to report.

## Conflicts of Interest

The authors declare no conflicts of interest.

## Data Availability

The data that support the findings of this study are available from the corresponding author upon reasonable request.
